# The Impact of Inequality: How to Make Sick Societies Healthier

**Published:** 2005-12-15

**Authors:** Magdalena Szaflarski

**Affiliations:** Institute for the Study of Health, Department of Family Medicine, University of Cincinnati, Cincinnati, Ohio

**Figure:**
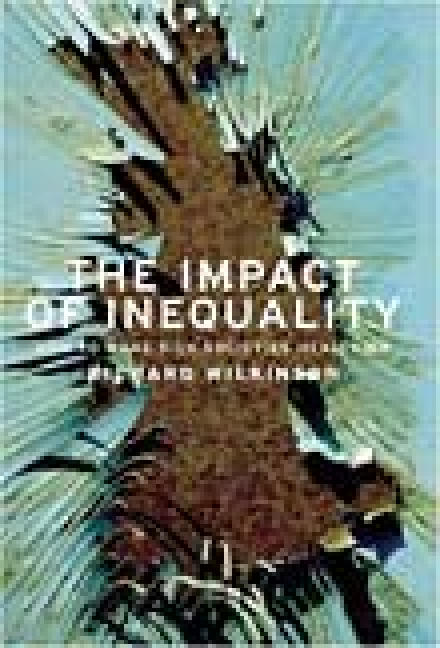


*The Impact of Inequality: How to Make Sick Societies Healthier*, by Richard G. Wilkinson, is a sequel to his book, *Unhealthy Societies: The Afflictions of Inequality* (1). The premise of both books is that the structure of social relations determines the health status of populations; that is, the greater the social inequality (e.g., income disparities) within a society, the poorer the health outcomes (e.g., the higher the death rates). Wilkinson's first book focused on documenting the relationship based on an emerging body of evidence; his second book takes the next step by reviewing the current state of knowledge, offering an explanation, and suggesting potential solutions to the problem. With its carefully assembled and weighted evidence and its clear and convincing argument, the second book is an excellent resource for social scientists, epidemiologists, public health officials, policy makers, and students.

This nine-chapter book addresses the primary questions of *how* and *why* inequality negatively affects individuals and populations. Chapters 2 through 4 describe the patterns of association between inequality and health and social outcomes, underscoring that "differences in inequality as small as those found between different market democracies or different U.S. states produce very substantial social and health effects." Chapter 2 reviews evidence supporting the idea that the quality of social relations is associated with income disparities, and Chapter 3 outlines psychosocial factors that contribute to ill health and premature death, including low social status, poor social affiliations, and negative childhood experiences, all of which can be linked to inequality. The data presented in Chapter 4 build upon previous chapters and strongly indicate that the more unequal a society is, the worse its health: "The pathway runs from inequality, through its effects on social relations and the problems of low social status and family functioning, to its impact on stress and health."

The remaining chapters explain the causal processes responsible for these relationships. In Chapter 5, the author uses violence as an example of a strong correlate of inequality and discusses the contributions of low social status and self-worth. Chapter 6 moves the theme forward and describes the social processes responsible for social distances and distinctions, including discrimination. In Chapter 7, race and gender inequality are examined, revealing among other things an interesting paradox: men appear to be more harmed by male domination than women are. Chapter 8 examines the pathway from the form of social organization (degree of inequality) through stress and coping mechanisms to physiological factors (e.g., cardiovascular, immune) that shape health status. Finally, in Chapter 9, the author frames the problem of inequality and health in terms of ideology and political objectives, revisiting the traditional democratic values: liberty, equality, and fraternity.

A systematic review of the existing research is one of the book's most important strengths. Wilkinson carefully reviews studies to date citing supporting evidence as well as negative findings. When comparing studies, he points out methodological variations and offers explanations for differences in findings. He chooses his examples with care, focusing on the most robust and convincing research; for example, he examines homicide as one type of violence and death rates as indicators of population health, definitions of which are similar across societies, allowing straightforward comparisons. The book also highlights current and high-priority social issues such as obesity and their links to social class throughout history.

In addition to the content, Wilkinson's book is well-organized and written in language that can reach a more general audience. What is somewhat unconventional is that some background information (e.g., evolution of human societies and social inequality) appears later in the book instead of at the beginning. However, this strategy seems to work well for this book — by presenting his argument and much of the supporting evidence in the first half of the book, Wilkinson succeeds in getting his point across more powerfully. There is one weakness to this scheme, however: Chapters 5 and 6 contain information and examples already introduced in previous sections, making these chapters somewhat redundant and less effective.

The success of the book is based in part on the author's obvious passion for the topic and his sincere concern about the social issue. To make his point, Wilkinson sometimes offers an extreme opinion or example, such as "perhaps we should liken the injustice of health inequalities to that of a government that executed a significant portion of its population each year without cause." Wilkinson's speculations provoke thought. He wonders, for example, how different the government's response to health disparities would be if the income gradient in health were opposite to the existing one: that is, if it were the higher income groups experiencing the worst health. Although written from a particular viewpoint — that of social justice and reform — the book does not attack capitalism per se, and it does not impose an extreme ideology. Instead, the author's view of social progress exists within the framework of modern society, where the market seems to be an inevitable element. Within that framework, inequality can be reduced, quality of social relations improved, and social stress decreased, all leading to improvements in health and well-being.

Wilkinson ends the book on an optimistic note: although differences in health inequalities across societies and history exist, change is possible, and inequalities can be reduced. As our moral universe expands along the line of democratic values and as we become more sensitive to the suffering and pain of others, the reduction of inequality and the improvement of well-being across the social strata should strengthen as political goals. This book is highly recommended to anyone interested in health and its related social issues.
